# Identification and characterization of a polyomavirus in the thornback skate (*Raja clavata*)

**DOI:** 10.1186/s12985-023-02149-1

**Published:** 2023-08-24

**Authors:** Joana Abrantes, Arvind Varsani, Paulo Pereira, Catarina Maia, Inês Farias, Ana Veríssimo, Fabiana Neves

**Affiliations:** 1 CIBIO-InBIO, Research Center in Biodiversity and Genetic Resources, University of Porto, Vairão, 4485-661 Portugal; 2 BIOPOLIS Program in Genomics, Biodiversity and Land Planning, CIBIO, Campus de Vairão, Vairão, 4485-661 Portugal; 3 Department of Biology, Faculty of Sciences, University of Porto, Porto, 4169-007 Portugal; 4 The Biodesign Center for Fundamental and Applied Microbiomics, Center for Evolution and Medicine, School of Life Sciences, Arizona State University, Tempe, AZ USA; 5 Structural Biology Research Unit, Department of Integrative, Biomedical Sciences, University of Cape Town, Observatory, Cape Town, 7925 South Africa; 6 Portuguese Institute for Sea and Atmosphere, Division of Modelling and Management of Fisheries Resources, Alges, 1495-165 Portugal

**Keywords:** Cartilaginous fishes, Thornback skate, *Polyomaviridae*

## Abstract

Members of the family *Polyomaviridae* have a circular double-stranded DNA genome that have been identified in various hosts ranging from mammals to arachnids. Here we report the identification and analysis of a complete genome sequence of a novel polyomavirus, *Raja clavata* polyomavirus (RcPyV1), from a cartilaginous fish, the thornback skate (*Raja clavata*). The genome sequence was determined using a metagenomics approach with an aim to provide baseline viral data in cartilaginous fish in different ecosystems. The RcPyV1 genome (4,195 nucleotides) had typical organization of polyomavirus, including early antigens (small T; Large T) encoded on one strand and late viral proteins (VP1; VP2) on the complementary strand. Maximum-likelihood phylogenetic analysis of the large T-antigen revealed that RcPyV1 clusters with a polyomavirus obtained from another cartilaginous fish, the guitarfish polyomavirus 1 (GfPyV1). These two share ~ 56% pairwise identity in LT and VP1 protein sequences. These analyses support the hypothesis that cartilaginous fishes have a specific lineage of polyomaviruses.

## Introduction

The family *Polyomaviridae* is composed of small, non-enveloped, double-stranded DNA viruses with a circular genome of approximately 4,000–7,000 nucleotides (nts) in length [1, 2]. These viruses exhibit a conserved organization with an early region and a late region separated by a non-coding regulatory region (NCRR) [3], which contains the early and late promoters and the origin of replication. The early region encodes up to five non-structural tumor antigen proteins, which are involved in viral replication and oncogenesis. The large and small tumor antigen proteins (LT and sT, respectively) are universally expressed by polyomaviruses [1, 3]. The LT is a multiregulatory protein that is required for the initiation of viral replication and activation of the late region promoter, but also for the suppression of its own promoter, thus regulating early gene expression (reviewed in [4]). The precise function of the sT protein is unclear, but it has been suggested to play a role in the regulation of the viral replication cycle (reviewed in [4]). The late region encodes the capsid proteins VP1, VP2 and VP3, which are important for virion assembly and nuclear egress [2, 3]. In addition, the polyomavirus simian virus 40 (SV40) also produces a late VP4 protein [5]. The early and late regions of polyomavirus (PyVs) genomes may further encode alternatively spliced forms of the encoded proteins, such as the alternative large T antigen open reading frame (ALTO; [6] or the agnoprotein [7]). The agnoprotein is a regulatory protein essential for sustaining a productive viral life cycle, being involved in viral DNA replication, viral transcription, virion maturation and release (reviewed in [4]). The ALTO protein has been shown to be expressed, but not being essential, during replication, most likely playing an accessory role [6]. More recently, the DUO protein has also been identified in different polyomavirus (https://ccrod.cancer.gov/confluence/display/LCOTF/Polyomavirus), however, there are no studies addressing its function.

PyVs have been identified in several mammals [8–11], birds [12], fishes [13, 14] and arthropods [15]. In fish, PyVs have been described in perciform fish such as the black sea bass (*Centropristis striata*) [16], the gilt-head sea bream (*Sparus aurata*) [17], the sharp-spined notothen (*Trematomus pennellii*) and emerald notothen (*Trematomus bernacchii*) [1, 14], but also in cartilaginous fish taxa such as the giant guitarfish (*Rhynchobatus djiddensis*) [1, 13]. Chimeric viral genomes that encode proteins related to those of PyVs have been reported in the eel species *Anguilla japonica* and *A. marmorata* [18–20]. Fish PyVs belong to at least two distinct evolutionary lineages, one comprising perciform-fish PyVs and the other encompassing cartilaginous fish PyVs [14], which only includes a single PyV detected in the giant guitarfish (GfPyV1; [13].

Cartilaginous fishes are the oldest group of extant vertebrates, being the most basal living jawed vertebrates. Cartilaginous fishes can be divided into two very distinct subclasses, Elasmobranchii (sharks, rays and skates) and Holocephali (chimaeras), which branched off from each other almost 420 million years ago [21]. They present a complex immune system, exhibiting one of the greatest functional diversities when compared to other vertebrates [22]. Screening for pathogen communities in cartilaginous fish hosts has been mostly opportunistic and descriptive, and has not covered the taxonomic and ecological diversity of the group. While only a few viruses have been isolated from cartilaginous fish taxa, the few data available show extensive retroviral diversity in the elephant shark (*Callorhinchus milii*) genome [23], and newly identified RNA and double stranded DNA (dsDNA) viruses in shark and ray species [13, 24–30].

Here, as part of an ongoing study aiming to provide a baseline data of the viruses associated with various cartilaginous fish with different ecologies, we report the identification of a complete polyomavirus genome from the thornback skate, *Raja clavata*, a coastal benthic elasmobranch from the order Rajiformes.

## Materials and methods

### Sampling

A total of ten *Raja clavata* individuals were collected during the Nephrops Survey Offshore Portugal survey (NepS (FU 28–29)) in June/July 2021 in R/V Mário Ruivo. This survey has been conducted yearly by the Portuguese Institute for the Sea and Atmosphere (IPMA), during the 2nd quarter (May-July), under the EU/DGMARE Fisheries’ Data Collection Framework (DCF), with the aim of monitoring the abundance and distribution of the main crustacean species, namely *Nephrops norvegicus* (Norway lobster), *Parapenaeus longirostris* (deepwater rose shrimp) and *Aristeus antennatus* (red shrimp) (ICES, 2016). The survey design follows a grid that covers the main crustacean fishing grounds in southwest and south coasts within the depth range of 200–750 m (ICES, 2022). The hauls are carried out during daytime at an average speed of 3.2 knots and the duration of each tow is 30 min.

### DNA extraction, Illumina sequencing and data processing

DNA was extracted from the spleen tissue from each of the ten *Raja clavata*. Briefly, approximately of 12 mg of tissue was homogenized with 300 µl of SM buffer (0.1 M NaCl, 50 mM Tris/HCl-pH 7.4, 10 mM MgSO_4_) and disrupted using a bioruptor. The homogenized sample was centrifuged at 10.000 rpm for 2 min and 200 µl of the supernatant was used to isolate viral DNA using the High Pure Viral Nucleic Acid Kit (Roche Diagnostic, USA), according to manufacturer’s specifications. The extracted viral nucleic acid was then enriched for circular DNA molecules using the rolling circle amplification (RCA) reaction with the TempliPhi™ kit (GE Healthcare, USA). The products from RCA were quantified using Qubit™ dsDNA HS Assay kit (Thermo Fisher Scientific, USA), pooled equimolarly, and sent to Macrogen Inc. (Korea) for library preparation (Nextera DNA XT) and sequencing on an Illumina Novaseq 6000. Following Illumina sequencing, the resulting pair-end-reads were trimmed using Trimmomatic [31], host genome sequences were removed using the RefSeq genome of the Rajiform *Amblyraja radiata* available at NCBI (RefSeq accession number GCF_010909765.2) as a reference with Bowtie2 [32]. The remaining reads were *de novo* assembled using Megahit v1.2.9 [33].

### Identification of viral genomes

The *de novo* assembled contigs were, in a first step, examined for putative viral matches using Diamond [34] against the NCBI RefSeq Virus database coupled with Cenote-Taker 2 [35]. The putative viral contigs with > 500 nucleotides in length and e-value ≤ 10^− 5^ were further analyzed with NCBI BLASTx/BLASTn searches against the refseq_protein and the nucleotide collection (nr/nt) databases, respectively.

### Viral genome analysis

Annotated fish and arachnids polyomavirus sequences were downloaded on March 2023 from https://ccrod.cancer.gov/confluence/display/LCOTF/Polyomavirus and were aligned using MAFFT under the algorithm L-INS-i [36].

RcPyV1 putative accessory proteins were annotated using Geneious software version 11.0.18 (https://www.geneious.com), by searching the genomic sequence for ORFs of at least 25 codons.

Pairwise nucleotide divergence calculations were performed for the two polyomaviruses isolated from cartilaginous fishes (GfPyV1 and RcPyV1) using the Sequence Demarcation Tool (SDT) version 1.2 in MUSCLE mode [37].

### Recombination analysis

The alignment was screened for recombination using RDP5 [38] with default settings. Only events with an associated p-value < 0.05 detected by three or more recombination detection methods implemented in RDP5 were accepted as plausible evidence of recombination.

### Phylogenetic analysis

The final dataset for the phylogenetic analysis included the polyomavirus identified in this study (RcPyV1) and the annotated polyomavirus sequences from fishes and arachnids retrieved from https://ccrod.cancer.gov/confluence/display/LCOTF/Polyomavirus. A total of nine protein sequences for LT and VP1 were aligned using the L-INS-i algorithm on MAFFT [36]. There were a total of 973 positions for LT and 895 positions for VP1 in the final aligned dataset. Maximum likelihood (ML) phylogenetic trees for LT and VP1 proteins were constructed using MEGAX [39]. The rtREV + G + I + F and WAG + G were used as the best-fit amino acid model for LT and VP1, respectively, as determined by MEGAX using ML as statistical method and the Bayesian information criterion as measure, and 1000 bootstrap replicates. The final ML trees were rooted on the arachnids clade.

## Results

### Polyomavirus genome analysis

**Fig. 1:**
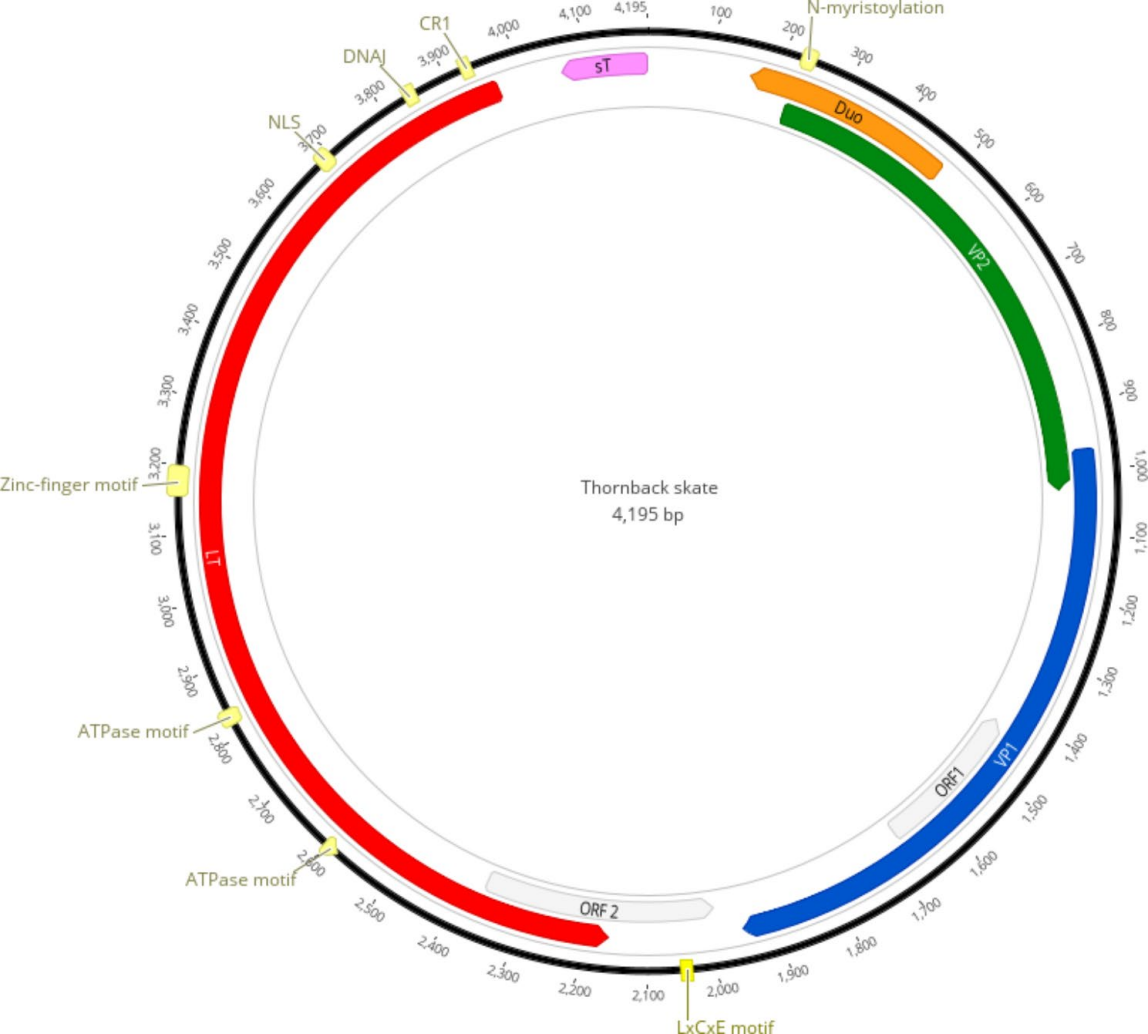
Genomic organization of the *Raja clavata* polyomavirus (RcPyV1). The size of the genome is indicated in base pairs (bp). The predicted small T antigen (sT) appears in pink, the Large T antigen (LT) is in red, the predicted major capsid protein (VP1) is in blue and the minor capsid protein (VP2) is in green. Additional ORFs of potential interest are indicated in light gray, and the Duo protein is in orange

A full genomic sequence of 4,195 nts was *de novo* assembled from the short read data. This genome has similarities to other polyomaviruses. Thus, based on the recommendation by the ICTV *Polyomaviridae* study group [40], the isolated viral genome was named *Raja clavata* polyomavirus 1 (RcPyV1, accession number OR159679). RcPyV1 presents the typical genomic organization of polyomaviruses (Fig. 1; Table 1), including the LT and sT antigen genes of the early region in one strand, and the VP1 and VP2 genes of the late region on the opposite strand.

**Table 1:** Predicted coding regions of RcPyV.

Gene	Start	Finish	Length (nts)	Direction	Size of product (aa)
Viral protein 2	224	1030	807	Forward	269
DUO protein	483	160	324	Reverse	108
Viral protein 1	696	1952	984	Forward	328
Putative ORF1	1673	1425	249	Reverse	83
Putative ORF2	2365	1994	372	Reverse	124
Large T antigen	3965	2160	1806	Reverse	602
Small T antigen	4195	4064	132	Reverse	44

The predicted LT antigen (1,806 nts; 602 aa) is encoded by a single ORF and contains several conserved motifs [41] such as the polyomavirus conserved region 1 (CR1) motif (LQKLL), N-terminal Dna J-like motif (HPDKGG), zinc binding domain (CVLCKEDKVHSETH) and the helicase domain with ATPase motifs (GPYNSGKT and GLCPVGLE) (Fig. 1 and Table 1). The putative sT antigen-like (132 nts; 44 aa) was identified 5’ of LT antigen; however, none of its conserved motifs were found and the BLAST searches retrieved no hit to any proteins in GenBank.

The predicted VP1 protein, which is the major structural protein (1952 nts; 328 aa), and the predicted VP2 protein (1030 nts; 269 aa) overlap by 62 nts, and VP2 encodes a predicted N-terminal myristoylation sequence (MGAALAV). We also identified the Duo protein (324 nts; https://ccrod.cancer.gov/confluence/display/LCOTF/Polyomavirus) and other ORFs of potential interest (Fig. 1; Table 2). Yet, we were not able to identify the regulatory Agnoprotein detected in the genome of the close relative giant guitarfish polyomavirus 1 (GfPyV1)[1].

**Fig. 2:**
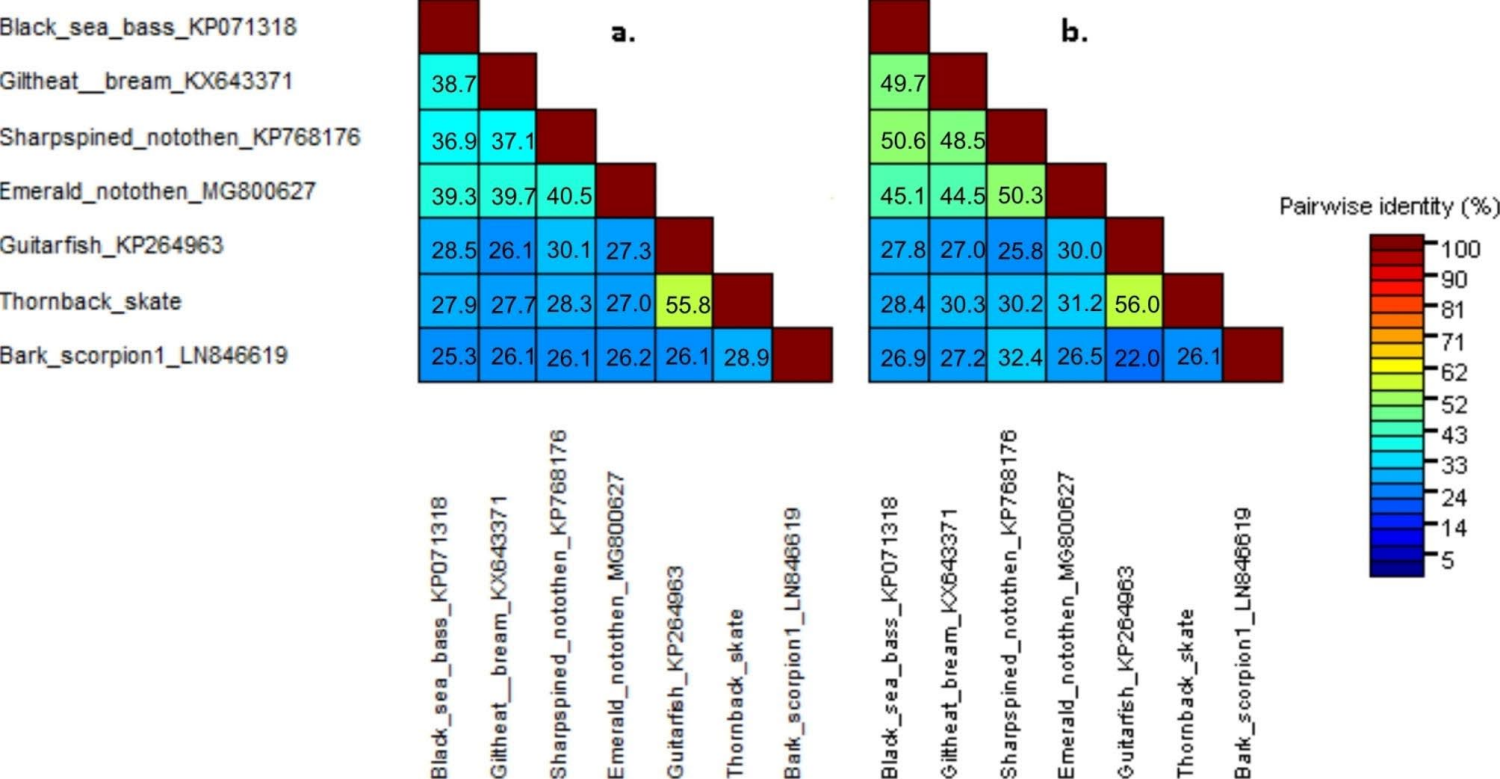
Percent identity between large T antigen (a) and VP1 (b) amino acid sequences of polyomaviruses from fish and arachnids. The figure was generated using SDT Virus Classification Tool

Pairwise comparisons of fish polyomaviruses using SDT showed that PyVs from the two cartilaginous fish share 55.8% and 56.0% of protein identity in LT and VP1 proteins, respectively, (Fig. 2). Among the other encoded viral proteins, the protein identities are lower (22.5% for sT, 38.2% for VP2 and 44.6% in DUO protein). When comparing cartilaginous fishes to perciform fish or arachnids, pairwise identities are lower (26.1–30.1% and 26.1–28.9%, respectively for LT and 25.8–31.2% and 22–26% for VP1, respectively).

**Table 2:** Conserved motifs identified in predicted proteins of RcPyV.

Protein	Name	Motif	Amino acid position
Viral protein 2	N-terminal myristoylation	MGAALAV	1–7
Putative ORF2	pRB1	LHCYE	106–110
Large T antigen	CR1	LQKLL	10–14
	DnaJ	HPDKGG	39–44
	NLS	PRRSIN	88–95
	Zinc-finger motif	CVLCKEDKVHSETH	257–270
	ATPase motif	GPYNSGKT	376–383
	ATPase motif	GLCPVGL	454–461

### Phylogenetic analysis

**Fig. 3:**
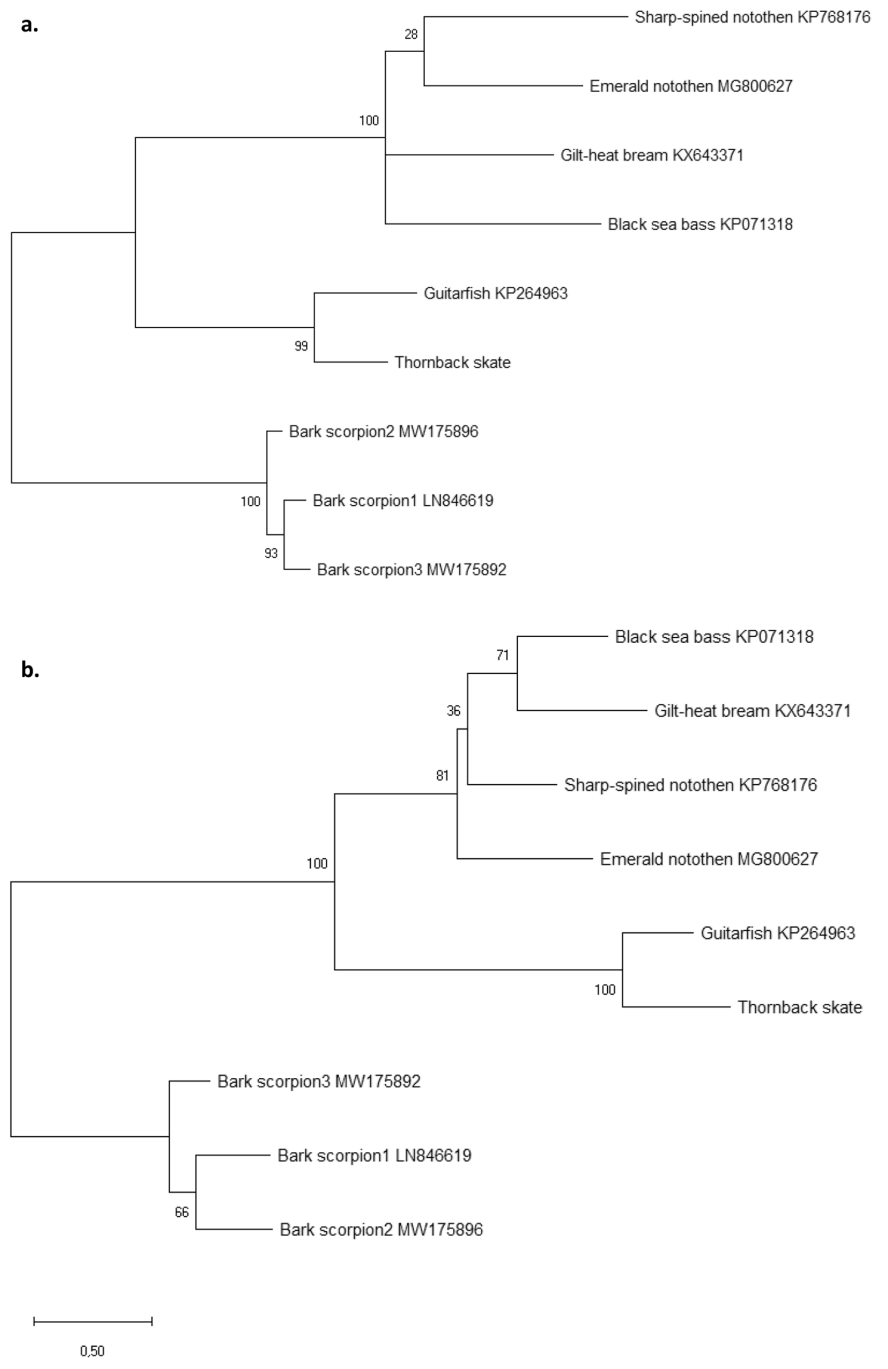
Maximum-likelihood phylogenetic trees of the **(a)** LT antigen and **(b)** VP1.

There were no recombination events detected between fish polyomaviruses. Maximum likelihood phylogenetic trees were constructed from LT (Fig. 3a) and VP1 (Fig. 3b) amino acid sequences and both phylogenetic trees show that GfPyV1 and RcPyV1 cluster together in a specific lineage, reflecting the host topology.

## Discussion

Polyomavirus genomes range from 3962 to 7369 nts, with the smallest PyV being identified in a cartilaginous fish, the giant guitarfish (*Rhynchobatus djiddensis*) [13]. The *R. clavata* polyomavirus reported here, RcPyV1, has a 4,195 nt-long genome, being the second smallest polyomavirus described to date.

Similar to other polyomaviruses, the RcPyV1 genome contains CDS homologous to sT, LT, VP1, VP2 and DUO encoding proteins (Fig. 1; Table 1). When compared with GfPyV1 [1], RcPyV1 lacks the Agnoprotein. The predicted sT antigen seems to be smaller (132 bp; 44aa) when compared to other vertebrates and, like in GfPyV1 [13], it does not contain any conserved motif. Previous studies on fish polyomaviruses indicates that sT usually presents the DnaJ motif while the remaining ORF show no similarity among sequences [14]. This is in agreement with our BLAST search results that revealed that RcPyV1 VP1 has no sequence identity to known proteins. In turn, the LT antigen, known to be crucial for viral replication [8], presents all the other conserved motifs described for polyomaviruses, with the exception of the retinoblastoma protein binding motif (pRB) which is specific to amniote polyomaviruses, and the protein phosphate 2 A (Fig. 1; Table 2). Within LT, we identified the CR1 motif (LQKLL), important for transcriptional regulation [42], the hexapeptide (HPDKGG) involved in protein interactions, the putative nuclear localization signal (PRRSIN), the zinc-finger motif (CVLCKEDKVHSETH) and ATPase motifs (GPYNSGKT and GLCPVGLE), which are important to recruit cellular proteins involved in replication [43].

Additional ORFs of potential interest were detected. These include the putative ORF1 that presents the LxCxE motif (LHCYE), the putative ORF2 and DUO protein. DUO protein has been identified in mammals, birds, fishes and aracnids (https://ccrod.cancer.gov/confluence/display/LCOTF/Polyomavirus), however its function and importance is still unkown.

According to ICTV Polyomavirdae study group recommendations for the classification of polyomaviruses, polyomaviruses that share < 85% pairwise identity in the LT antigen should be considered as a separate species [40]. The LT antigen sequences from the two cartilaginous fish share 56% identity and thus GfPyV1 and RcPyV1 represent members of two different species of polyomaviruses. However, fish polyomaviruses have not yet been assigned to a genus [15].

No signs of infection were detected in the collected samples, however, since this virus was detected in the spleen, it is likely that RcPyV1 is not a contaminant from the environment, but rather a virus infecting the thornback skate. This is also supported by the previous identification of GfPyV1 in the giant guitarfish [13], which is phylogenetically related to RcPyV1.

It has been proposed that polyomaviruses evolution is driven by their hosts, although they often show higher among-sequence divergence levels compared to those of their hosts suggesting that other factors contribute to their evolution [1, 9]. Previous analysis [14] suggested that fish polyomaviruses are divided in two lineages: one lineage clustering viral genomes isolated from perciform fish and lacking the DnaJ domain, and the other lineage grouping viral genomes isolated from cartilaginous fish exhibiting the DnaJ domain. Our results are in line with these observations as in both phylogenetic trees, GfPyV1 and RcPyV1 cluster together, but separately from the remaining perciform fish PyVs. Recombination, which plays an important role in the evolution of PyVs [1, 14], was not found in the GfPyV1 and RcPyV1.

In conclusion, we identified a novel polyomavirus from the cartilaginous fish *Raja clavata*, which presents the typical features of polyomaviruses: LT with conserved motifs, sT, VP1 and VP2 proteins. RcPyV1 belongs to the same evolutionary lineage as the previously identified GfPyV1, reinforcing that Perciform and cartilaginous fish PyVs are not monophyletic, but rather represent two divergent groups. The pairwise comparisons between RcPyV1 and GfPyV1 and the remaining fish PyVs are in line with this.

## Data Availability

The sequence reported here is available in NCBI with the accession number OR159679.
